# Exploration of the role of the penicillin binding protein 2c (Pbp2c) in inducible β-lactam resistance in *Corynebacteriaceae*

**DOI:** 10.3389/fmicb.2024.1327723

**Published:** 2024-05-09

**Authors:** Marie Lavollay, Céline Buon, Vincent Le Moigne, Fabrice Compain, Armel Guyonvarch, Matthieu Fonvielle

**Affiliations:** ^1^INSERM, Université Paris Cité, Sorbonne Université, Centre de Recherche des Cordeliers, Paris, France; ^2^Service de Microbiologie, Hôpital Européen Georges Pompidou, AP-HP Centre, Université Paris Cité, Paris, France; ^3^Institut Mutualiste Montsouris (IMM), Service de Microbiology, Paris, France; ^4^Université Paris-Saclay, UVSQ, Inserm, Infection et Inflammation, Montigny-le-Bretonneux, France; ^5^Institute for Integrative Biology of the Cell (I2BC), CEA, CNRS, Université Paris-Saclay, Gif-sur-Yvette, France

**Keywords:** *Corynebacteriaceae*, β-lactam, Pbp, Pbp2c, *Corynebacterium jeikeium*, *Corynebacteria*

## Abstract

Six genes encoding putative high molecular weight penicillin-binding proteins (Pbp) are present in the genome of the β-lactam-resistant strain *Corynebacterium jeikeium* K411. In this study, we show that *pbp2c*, one of these six genes, is present in resistant strains of *Corynebacteriaceae* but absent from sensitive strains. The molecular study of the *pbp2c* locus from *C. jeikeium* and its heterologous expression in *Corynebacterium glutamicum* allowed us to show that Pbp2c confers high levels of β-lactam resistance to the host and is under the control of a β-lactam-induced regulatory system encoded by two adjacent genes, *jk0410* and *jk0411*. The detection of this inducible resistance may require up to 48 h of incubation, particularly in *Corynebacterium amycolatum*. Finally, the Pbp2c-expressing strains studied were resistant to all the β-lactam antibiotics tested, including carbapenems, ceftaroline, and ceftobiprole.

## Introduction

The genus *Corynebacterium* includes bacteria with very different lifestyles and metabolic abilities (Funke et al., 1997). Certain species, such as *Corynebacterium diphtheriae*, are human pathogens and others opportunistic pathogens, whereas *Corynebacterium glutamicum*, a non-pathogenic species, is of great biotechnological importance for the industrial production of amino acids (Hermann, 2003; Liu et al., 2023). Among opportunistic pathogens, *Corynebacterium jeikeium* and *Corynebacterium urealyticum* are components of human skin flora. *C. jeikeium* belong to the lipophilic *Corynebacteriaceae* sub-family and is a nosocomial pathogen commonly involved in catheter infections, prosthetic endocarditis (Rezaei Bookani et al., 2018; Gupta et al., 2021), periprosthetic joint infections (Seutz et al., 2023), and sepsis (Bernard, 2012), whereas *C. urealyticum* is mostly involved in urinary tract infections, such as alkaline cystitis (Soriano et al., 1985), granulomatous mastitis (Lu et al., 2023), and post-kidney transplant pyelitis (Soriano and Tauch, 2008). The diagnosis and treatment of *Corynebacteriaceae* can be complicated by the inability to distinguish colonization from contamination and infection, and by the high prevalence of multidrug resistance in clinical isolates, especially to β-lactams, the most widely used class of antibiotics (Soriano et al., 1995; Fernandez-Roblas et al., 2009; Bluth et al., 2015). For example, the minimal inhibitory concentration (MIC) for penicillin has increased from 0.025 to over 256 mg/L for the most resistant *Corynebacterium* species and strains (Fernandez-Roblas et al., 2009).

The general cell envelope architecture and synthesis in *Corynebacteriaceae* is well documented (Burkovski, 2013; Levefaudes et al., 2015). Even if some minor differences exist between different species, the cell envelope is an atypic multilayered one. This envelope is made, from the inner part to the outer part, of a cytoplasmic membrane, a peptidoglycan layer, a connecting glycan layer and an external mycomembrane. The cytoplasmic membrane is an asymetric phospholipid bilayer mainly composed of phosphatidylglycerol and phosphatidylinositol, with palmitic acid (16:0) and decanoic acid (18:1) as dominating fatty acids. Lipomannan and lipoarabinomannan polymers are covalently bound to phospholipids from the outer sheet of the phospholipid bilayer and anchored in the murein sacculus. Overlaying this cytoplasmic membrane is a thin peptidoglycan of the A1γ type, with a glycan part made of alternating β-1,4-linked *N*-acetylglucosamine and *N*-acetyl muramic acid units. Cross-linking of glycan polymers occur via direct peptide bounds, without interpeptide bridges. The major peptides found in mature peptidoglycan in *Corynebacteriaceae* are the tetrapeptide L-Ala-γ-D-Glu-meso-DAP-D-Ala and the tripeptide L-Ala-γ-D-Glu-meso-DAP. In *C. jeikeium*, the α-carbonyl group of D-Glu and the ε-carbonyl group of meso-DAP residues are partially amidated (Lavollay et al., 2009; Levefaudes et al., 2015).

In *Corynebacteriaceae*, peptide bridges occur either between meso-DAP and D-Ala (4–3 cross-links) or between two meso-DAP residues (3–3 cross-links), indicating the participation of D,D- and/or L,D-transpeptidases. In *C. jeikeium*, final steps of peptidoglycan reticulation is mediated by five high molecular weight class A and B penicillin binding proteins (Pbp), responsible for 62% of cross links and two L,D-transpeptidases (Ldt) responsible of the remaining 38% cross links (Lavollay et al., 2009).

The outer part of the envelope in *Corynebacteriaceae*, with the noticeable exception of *C. amycolatum*, is made of an additional barrier, the mycomembrane. In *Corynebacteriaceae*, peptidoglycan is connected to the mycomembrane by complex glycan polymers. These polymers are made of arabinogalactan (D-arabinose and D-galactose containing multimers), attached to linker units made of galactose, rhamnose and *N*-acetylglucosamine, linker units and arabinogalactan are covalently attached to *N*-acetyl muramic acid. The inner sheet of mycomembrane is made of corynomycolic acids (*circa* 30 carbon atoms) covalently linked to arabinogalactan and free corynomycolic acids. The outer sheet of the mycomembrane is mainly made of trehalosyl mono- or di-mycolates. Last, in some *Corynebacteriaceae*, an outer crystalline surface layer composed of a single protein could exist (Burkovski, 2013).

A crucial step in envelope formation is the transpeptidation step of murein. A failure of transpeptidation leads to cell disruption and cell death due to the internal osmotic pressure. Even if Pbp and Ldt proteins catalyze the formation of similar amide bonds, they are not structurally related and use different substrates. Pbp are serine active enzymes that use the disaccharide-L-Ala-γ-D-Glu-meso-DAP-D-Ala-D-Ala-pentapeptide as the substrate while Ldt are cysteine active enzymes using the disaccharide-L-Ala-γ-D-Glu-meso-DAP-D-Ala-tetrapetide as the susbtrate. Although L,D-transpeptidases are ampicillin-insensitive cross-linking enzymes (Mainardi et al., 2007; Lavollay et al., 2009), they are unlikely to directly contribute to the resistance to this antibiotic in *C. jeikeium*, the formation of their tetrapeptide substrate being dependent of the low molecular weight Pbp D,D-carboxypeptidase (Pbp4_Cjk_), inactivated by ampicillin (Lavollay et al., 2009). The *C. jeikeium* β-lactam resistance may also result from other factors such as the presence of β-lactamases, low affinity Pbp, or the impermeability of the cell wall. In this study, we investigated the putative role of two genes from *C. jeikeium* (*jk0411* and *jk0412*), only found in strains with significant β-lactam resistance and encoding two proteins with strong analogies to β-lactamases and Pbp, respectively. Six genes encoding putative high-molecular-mass Pbp were previously identified in the genome of the *C. jeikeium K411* resistant strain (Lavollay et al., 2009). Only one of these genes, *jk0412*, predicted to encode a high-molecular class B Pbp, was found in the genome of *C. jeikeium* resistant strains but absent in susceptible strains (Lavollay et al., 2009).

To directly show the contribution of *pbp2c* to β-lactams resistance in *C. jeikeium*, the deletion of the *pbp2c* gene in *C. jeikeium* would have been the ideal method. Unfortunately, this was not possible. Although genetic tools for gene deletion in *C. glutamicum* have been published (Schafer et al., 1994; Hu et al., 2013) they could not be applied to *C. jeikeium* because of several technical limitations, such as no highly efficient transformation system available (Barzantny et al., 2013), no possibility to use the counter selecting *sacB* gene for pK18*mobsac*-mediated edition (Dusch et al., 1999), because of the lack of any PTS system (Tauch et al., 2005), no replicative plasmid in *C. jeikeium,* with a thermosensitive origin of replication for CRE/LOX edition (Hu et al., 2013). Moreover, *C. jeikeium* is resistant to a wide range of antibiotics, with MIC over 128 μg/mL for the antibiotics commonly used in molecular biology, namely kanamycin, tetracycline, chloramphenicol, and ampicillin (Rosato et al., 2001). Therefore, because of all these limitations, we decided to study the potential implication in β-lactam resistance of the *pbp2c* gene from *C. jeikeium* in a heterologous system based on *C. glutamicum*, a species taxonomically close to *C. jeikeium*, highly sensitive to most of β-lactams, and for which all the molecular tools needed for this study are available and functional.

We also report here that all resistant *C. jeikeium* strains tested possess two additional genes, *jk0410* and *jk0411*, located upstream of the *pbp2c* gene, and that, one or both, could be involved in the regulation of the expression of *pbp2c*. Studying the effect of various β-lactams on the induction phenomena highlighted that current European Committee on Antimicrobial Susceptibility Testing (EUCAST) recommendations for detecting β-lactam resistant strains, which do not take into account such inducible resistance, could lead to poorly adapted choices for detection and treatment of infections due to *Corynebacteriaceae*.

## Materials and methods

### Bacterial strains and growth conditions

The bacterial strains used in this study are listed in Supplementary Table S1. *Escherichia coli* and *C. glutamicum* were grown in Brain Heart Infusion media (BHI, Difco) at 37°C under agitation (180 rpm). Reference strains *C. jeikeium* CIP82.51 (CjkS), CIP103337 (CjkR), and *C. urealyticum* DSM7109 (CuR) were grown in BHI medium supplemented with 0.5% Tween 80 (Sigma-Aldrich). Antibiotics used for selection were ampicillin (Ap) at 100 μg/mL (Euromedex, Souffelweyersheim, France), kanamycin (Km) at 50 μg/mL (Duchefa, Haarlem, The Netherlands), and chloramphenicol (Cm) at 30 μg/mL for *E. coli* and 10 μg/mL for *C. glutamicum* (Duchefa).

### Susceptibility testing

Reference strains and clinical isolates from invasive specimens (hemocultures, deep purulence) were studied in accordance to the recommendations of the Antibiogram Committee of the French Society of Microbiology Version 2.02019 (CA-SFM) using the disk diffusion, broth microdilution, and E-test methods.

For the disk diffusion assay, susceptibility testing was performed on Mueller-Hinton agar supplemented with 5% defibrinated horse blood and 20 mg/L β-NAD (MH-F, BioMérieux, La Balme-Les-Grottes, France) with an inoculum of 0.5 McFarland units. Plates were incubated under 5% CO_2_ at 35 ± 2°C. The inhibition zones were determined after 24 h of incubation. If a reduction (>2 mm) of the inhibition zone was detected after an additional incubation of 24 h, the diameters observed at 48 h are indicated in brackets (Table 1). Antibiotic disks were purchased from Oxoid (Basingstoke, England) or prepared extemporaneously by loading 10 μg potassium clavulanate (Sigma-Aldrich) onto 6-mm disks (Dutscher, Brumath, France).

**Table 1:** Sensitivity of *Corynebacterium* strains determined by the disk diffusion assay.

		Diameter of inhibition zones (mm) for strains [*pbp2c* gene]							
		*C. jeikeium*			*C. urea*^b^	*C. glutamicum*^c^			
Antibiotic	Disk load^a^	CjkS [none]	CjkR [*jk0412*]	K411 [*jk0412*]	CuR [*Cu1571*]	RES167 [none]	#3278 [none]	#3279 [*jk0412*]	#3322 [*Cu1571*]
**β-Lactam**									
*penam*									
Benzylpenicillin	1	10	n	n	n	23	23	N	n
Ampicillin	2	15	n	n	n	27	27	N	n
Ampicillin	10	32	n	n	n	35	35	13	13
Amoxicillin/clavulanate	20/10	35	n	n	n	40	40	22	22
Ticarcillin	75	34	n	n	n	34	34	22	19
Ticarcillin/clavulanate	75/10	34	n	n	n	34	34	22	19
Piperacillin	30	22	n	n	n	27	27	12 (6)	10 (6)
Piperacillin/tazobactam	30/6	22	n	n	n	32	32	12 (6)	10 (6)
Temocillin	30	n	n	n	n	10	10	N	n
*cephem*									
Cefalexin	30	25	n	n	n	35	35	17 (n)	17 (n)
Cefoxitin	30	25	21 (n)	15 (n)	n	35	35	16 (n)	16 (n)
Cefotaxime	5	18	8 (n)	n	n	26	26	10 (n)	10 (n)
Cefepime	30	30	28 (n)	n	n	35	35	12 (n)	12 (n)
Ceftaroline	5	30	15 (n)	n	n	37	37	13 (n)	13 (n)
Ceftazidime	10	n	n	n	n	9	9	N	n
*Monobactam*									
Aztreonam	30	n	n	n	n	n	n	N	n
*carbapenem*									
Ertapenem	10	35	26 (20)	15 (n)	n	40	40	13 (n)	12 (n)
Imipenem	10	35	n	n	n	40	40	29 (25)	29 (25)
Meropenem	10	35	25 (n)	n	n	40	40	20 (n)	20 (n)
Clavulanate	10	n	n	n	n	15	15	N	n
*Other*									
Vancomycin	5	20	20	20	20	20	20	20	20
Teicoplanin	30	19	19	19	19	19	19	19	19
Gentamicin	15	25	n	n	n	31	31	31	31
Norfloxacin	10	22	22	12	22	24	24	24	24
Erythromycin	15	35	26	13	12	25	25	25	25
Clindamycin	2	20	n	n	n	13	13	13	13
Pristinamycine	15	32	36	36	36	30	30	30	30
Tetracycline	30	31	n	21	16	31	31	31	31
Chloramphenicol	30	30	10	10	10	30	17	17	17

The inhibition zones were determined after 24 h of incubation. If a reduction (>2 mm) of the inhibition zone was detected after an additional incubation of 24 h, the diameters observed at 48 h are indicated in brackets. n, no inhibition zone, as bacteria grew at the contact of the disks, which had a diameter of 6 mm. Values are the mean of at least three determinations. ^a^The antibiotic quantity loaded on disks is indicated in μg except for benzylpenicillin (units). ^b^*C. urea* = *C. urealyticum*. ^c^*C. glutamicum* *#3278, #3279, and #3322* are derivatives of strain RES167 harboring plasmids pMM36, pMM36ΩP*_trc_-jk0412* (pML2), and pMM36ΩP*_trc_-cu1571*, respectively.

Broth microdilution assays were performed according to CA-SFM V 2.02019 guidelines in cation-adjusted Müller-Hinton II broth (BD-Difco, Le Pont-de-Claix, France) supplemented with 5% lysed defibrinated horse blood (Oxoid, Basingstoke, England) and 20 mg/L β-NAD (Sigma-Aldrich). The inoculum containing 5 × 10^5^ CFU/mL in a fresh medium was prepared from an overnight 10 mL preculture under agitation. 96-well plates containing 100 μL were incubated aerobically at 35 ± 2°C for 24 and 48 h. The microtiter plates contained twofold serial dilutions of penicillin G (Panpharma, Boulogne-Billancourt, France), meropenem (AstraZeneca, Rueil-Malmaison, France), or potassium clavulanate (Sigma-Aldrich) from 1,024 to 0.0625 μg/mL. The MICs of penicillin G and meropenem were also determined in combination with a fixed concentration (10 μg/mL) of potassium clavulanate.

E-test assays were performed on MH-F medium according to the recommendations of the manufacturer of the antibiotic-containing strips. Benzylpenicillin, amoxicillin, imipenem, and meropenem MIC Test Strips were provided by BioMérieux (Craponne, France). Ceftaroline and ceftobiprole MIC Test Strips were provided by Liofilchem (Roseto degli Abruzzi, Italy). Benzylpenicillin and meropenem were also tested in association with clavulanate (10 μg/mL) which was added in the media. Plates were incubated under 5% CO_2_ at 35 ± 2°C for 24 and 48 h.

### Preparation of genomic DNA from *Corynebacteriaceae*

Bacteria from a 24 h culture (2 mL) were harvested by centrifugation (7,500 ×*g* for 3 min at 4°C), resuspended in 1 mL 25 mM Tris-Cl (pH 8.5) containing 10 mM EDTA, 1% glucose, and 20 mg/mL lysozyme, and incubated at 37°C for 2 h. DNA was extracted using the Wizard Genomic DNA Purification Kit (Promega, Charbonnières-les-Bains, France) or an Ingenius apparatus (ELITechGroup, Puteaux, France) according to the manufacturers’ instructions.

### Detection of *pbp2c* by polymerase chain reaction

The primers used for *pbp2c* detection are described in Supplementary Table S2. The detection of *pbp2c* by PCR was carried out in 20 μL of a reaction mixture containing 1 μL genomic DNA extracted from the bacteria, 200 μM of each dNTP, 24 pmol of each primer, 2 units of VENT DNA polymerase (NEB, Evry, France), and 2 μL 10× Thermopol buffer (NEB). The reaction was carried out for 35 cycles according to the following program: denaturation at 95°C for 30 s and annealing and primer extension at 72°C for 90 s. After PCR amplification, 5 μL of the reaction mixture was loaded onto a 1.0% agarose gel containing ethidium bromide (0.5 μg/mL). After electrophoresis, the band of amplified DNA (1,401 bp) was visualized under UV light (254 nm).

### Heterologous expression of *pbp2c* genes in *Corynebacterium glutamicum*

The *pbp2c* genes from *C. jeikeium* (*jk0412*) and *C. urealyticum* (*cu1571*) were amplified using the primers Fwjk0412_1-20 L and Revjk0412_1782–1762 (Supplementary Table S2) and cloned into the vector pBlunt to give pBluntΩ*jk0412* and pBluntΩ*cu1571*, *respectively*. The *pbp2c* genes were subcloned into the vector pET2818 using NcoI and XhoI (NEB) (pET2818Ω*jk0412* and pET2818Ω*cu1571*). The *trc* promoter (*P_trc_*) from plasmid pKK388-1 (Brosius, 1988) was amplified using the primers Ptrc_Fw_4980 and Ptrc_Rev_353 and cloned into pBlunt. A 438-bp NcoI-EcoRV fragment from the resulting plasmid pBluntΩ*P_trc_* was cloned upstream of the *pbp2c* genes of pET2818Ω*jk0412* and pET2818Ω*cu1571* to generate the pET2818Ω*Ptrc*-*jk0412* and pET2818Ω*Ptrc-cu1571* plasmids. For the latter cloning step, pET2818 derivatives were digested by BglII, treated with the Klenow fragment of DNA polymerase I (NEB), and digested with NcoI. The plasmids pET2818Ω*P_trc_*-*jk0412* and pET2818Ω*P_trc_*-*cu1571* were digested with BamHI and XhoI and the 2,119-bp fragment carrying the *P_trc_*-*pbp2c* fusions cloned into the *E. coli* - *C. glutamicum* shuttle vector pMM36 (Merkamm et al., 2003). The resulting plasmids pMM36Ω*P_trc_*-*jk0412* and pMM36Ω*P_trc_*-*cu1571* (Supplementary Table S3) were introduced by electro-transformation into *C. glutamicum* RES167 as previously described (Bonamy et al., 1990).

### Production and purification of recombinant proteins

A fragment of the *pbp2c* gene from *C. jeikeium* K411 (*jk0412*) encoding Pbp2c without the N-terminal transmembrane segment (residues 32–593) was amplified using the primers Fwjk0412_93-113S and Revjk0412_1782-1762 (Supplementary Table S2) and cloned into the vector pET-TEV using the NdeI and XhoI restriction sites (underlined in Supplementary Table S2). pET-TEV is a modified version of pET28a in which the thrombin cleavage site has been replaced by a TEV cleavage site and the T7 tag deleted (Supplementary Table S3) (Houben et al., 2007). The resulting plasmid pET-TEVΩ*jk0412* encodes the Pbp2c protein with a six-histidine tag at its N-terminus followed by a TEV cleavage site (MGSSHHHHHHSSGENLYFQGHM). Expression of the protein was carried out in *E. coli* BL21(DE3) harboring pET-TEVΩjk0412. The bacteria were grown at 37°C in three liters of BHI broth supplemented with kanamycin (50 μg/mL). Expression was induced at 16°C for 17 h by adding 0.5 mM isopropyl-β-D-thio-galactopyranoside (IPTG) when the OD_600_ reached 0.7. The His-tagged Pbp2c protein was purified from a clarified lysate by metal affinity and size exclusion chromatography. Briefly, the crude extract from a 3 L culture of *E. coli* BL21(DE3) harboring pET-TEVΩjk0412 was clarified by centrifugation (16,600 ×*g* for 30 min at 4°C). The His-tagged-Pbp2c protein was purified from the clarified lysate by affinity chromatography on Ni^2+^-nitrilotriacetate-agarose resin (Qiagen GmbH, Hilden, Germany) and elution with 25 mM Tris-Cl (pH 7.5) containing 300 mM NaCl and imidazole at 50, 100, and 250 mM. His-tagged-Pbp2c-containing fractions were identified by 12% SDS-PAGE, pooled, and loaded onto a SuperDex® 200 HL 26/60 column (Cytiva, Uppsala, Sweden) equilibrated with 25 mM Tris-Cl (pH 7.5) containing 300 mM NaCl. The His-tagged-Pbp2c protein was eluted as a monomer according to the calibration curve of the column. The concentrated protein was stored at −20°C in 25 mM Tris-Cl buffer (pH 7.5) containing 300 mM NaCl and complemented with 50% glycerol (v/v). Protein purity was assessed by 12% SDS-PAGE and analyzed by electrospray mass spectroscopy in the positive mode after buffer exchange on a Hitrap®-Desalting column (Cytiva) previously equilibrated in MES buffer (100 mM, pH 6.4). The observed mass of His-tagged-Pbp2c was in agreement with the mass deduced from the Pbp2c sequence without the N-terminal residue Met (calculated: 62,974 Da; observed: 62,975 Da).

The *jk0411* gene from *C. jeikeium* K411 encoding Bla_Cor_ was amplified using the primers jk0411_Fw_XbaI and jk0411_Rev_NotI (Supplementary Table S2) and cloned into the pET-TEV as an XbaI-NotI DNA fragment (restriction sites underlined in Supplementary Table S2). Mutagenesis of the *jk0411* gene to obtain Bla_Cor_ S45A was performed using the QuikChange® site-directed Mutagenesis kit according to the user’s manual (Agilent Technologies). Expression and purification of Bla_Cor_ and Bla_Cor_ S45A were performed as described for the His-tagged-Pbp2c protein. The observed masses of Bla_Cor_ and Bla_Cor_ S45A were in agreement with the masses deduced from the protein sequences without the N-terminal residue Met (32,825 and 32,809 Da, respectively).

### Growth curves

*Corynebacteriaceae* species and strains were cultivated overnight in 10 mL BHI-Tween 80 (0.5%) medium in the presence (induced) or absence (uninduced) of a sub-inhibitory concentration (20 μg/mL) of ampicillin or imipenem. Cultures were diluted to an OD_600_ of 0.1 in the same media (50 mL) and incubated at 37°C with shaking. When the OD_600_ reached 0.4, meropenem was added to each culture to a final concentration of 32 μg/mL and the OD_600_ were monitored for an additional 26 h duration.

### Detection of Pbp2c-β-lactam adducts by electrospray mass spectrometry

The formation of enzyme-drug adducts between purified Pbp2c and various β-lactams were tested by incubating the protein (20 μM) with a 10-molar excess (200 μM) of antibiotics (amoxicillin, cefoxitin, imipenem, meropenem, ampicillin) for 1 h at 20°C in MES buffer at pH 6.4 (100 mM) and were stored at −20°C prior to mass spectrometry analysis. Protein and protein-β-lactam adduct masses were detected by electrospray mass spectrometry as previously described (Mainardi et al., 2007). Briefly, 5 μL of each reaction mixture was mixed extemporaneously with 5 μL acetonitrile and 1 μL 1% formic acid. The mixture was injected directly into the mass spectrometer (Qstar Pulsar I, Applied Biosystems) in positive mode at a flow rate of 50 μL/min using a buffer containing acetonitrile (50%) and formic acid (0.5%).

### Preparation of protein extracts

Bacteria were grown aerobically for 24 h in BHI medium supplemented with 0.5% Tween 80, harvested by centrifugation and resuspended in 100 mM Tris-Cl buffer (pH 7.0) to a turbidity equivalent to 25 OD_600_ units. This experiment was also performed using the same medium, but containing ampicillin (20 μg/mL) or clavulanate (10 μg/mL). Bacteria were successively washed, suspended in 100 mM Tris-Cl buffer (pH 7.0), and lysed by FastPrep with 0.17 μm glass beads (3 × 30 s, Speed 6.5; QBIOgene, Illkirch, France). Protein concentrations were determined using the Bio-Rad Bradford protein assay according to the manufacturer’s instructions, with bovine serum albumin as the standard.

### Mouse anti-Pbp2c antibodies

Purified His-tagged-Pbp2c was subcutaneously injected into five BALB/c mice (Janvier, France) (20 μg per mouse) with incomplete Freund’s adjuvant (1/1, v/v) on days 1, 28, and 57. One week after days 28 and 57, blood samples were obtained from the retroorbital plexus, centrifuged, and stored at −20°C until use. Mouse experiments were performed according to institutional and national ethical guidelines (Agreement n°783,223; approved by the Ministry of Higher Education and Research with APAFIS#11465-2016111417574906v4).

### Western blot analysis

Proteins from crude extracts (10 μg) were separated on 15% SDS-PAGE, transferred to a nitrocellulose membrane (Hybond, Amersham Biosciences, Little Chalfont, United Kingdom), and incubated with mouse anti-Pbp2c antiserum (1/5,000). Nitrocellulose membranes were incubated in 10 mM Tris-Cl buffer pH 7.5 containing 150 mM NaCl, 0.025% Tween 20, and 2.5% nonfat dry milk for 1 h at 4°C. Peroxidase-conjugated goat anti-mouse antibodies (Life Technologies, Saint-Aubin, France; 1/6,000) were used as secondary antibodies. Pbp2c-antibodies complexes were detected by chemiluminescence (Amersham ECL Western Blotting Detection Kit, Cytiva) using a film exposure time of 5 min. Purified His-tagged-Pbp2c (3 ng) was used as the positive control.

### β-Lactamase activity

β-lactamase activity was assessed by following the hydrolysis of the chromogenic β-lactamase substrate nitrocefin. The assay mixture contained 500 μM nitrocefin (Sigma-Aldrich) and 50 μg protein extract in a final volume of 50 μL of 10 mM phosphate buffer (pH 6.5). Hydrolysis of nitrocefin was monitored spectrophotometrically at 486 nm for 30 min at room temperature (Δε = 15,200 M^−1^ cm^−1^). Protein extracts of *C. jeikeium* CIP82.51 and *Mycobacterium chelonae* #2445 (generous gift from B. Heym, Institut Pasteur, France) were used as negative and positive controls, respectively.

## Results

### Heterologous expression of the *Corynebacterium jeikeium* and *Corynebacterium urealyticum pbp2*c genes in *Corynebacterium glutamicum* induce β-lactam resistance

Our first objective was to determine whether the production of Pbp2c was sufficient for β-lactam resistance in a sensitive *Corynebacterium* host. To obtain Pbp2c production, the *pbp2c*-encoding genes from *C. jeikeium* (*jk0412*) and *C. urealyticum* (*cu1571*) were cloned under the control of the *trc* promoter (*P_trc_*) into *E. coli* prior to transfer into the sensitive *C. glutamicum* strain RES167 using the shuttle transfer plasmid pMM36 (Merkamm et al., 2003). The sensitivity of *Corynebacterium* strains was determined by disk diffusion assay (Table 1), microdilution assay (Table 2), and E-Test assay (Table 3). The *C. glutamicum* RES167 strain does not contain any class B Pbp-encoding gene related to *pbp2c* (Lavollay et al., 2009) and, according to the PK/PD non species related breakpoints of the CA-SFM V2.0 2019, is sensitive to all tested β-lactam antibiotics, with the exception of aztreonam, ceftazidime, and temocillin, known to be inactive against Gram-positive bacteria (Table 1). To ensure that Pbp2c production did not affect the *C. glutamicum* growth, due to a potential toxicity, we compared the growth of *C. glutamicum* strains #3278 and #3279 harboring either the empty vector pMM36 or the pMM36Ω*P_trc_*-*jk0412* vector (pML2), respectively. No significant difference was observed between these two strains (Supplementary Figure S1). The activity of drugs belonging to glycopeptides, aminoglycosides, fluoroquinolones, macrolides and related, tetracycline and chloramphenicol was not altered by expression of the *pbp2c* genes (Table 1). Although the introduction of the empty pMM36 vector (Supplementary Table S3) did not alter the sensitivity of RES167 to antibiotics (Strain #3278 in Table 1), constitutive expression of the *pbp2c* genes from *C. jeikeium* and *C. urealyticum* in *C. glutamicum* resulted in large decreases (at least 12 mm) in the diameter of the inhibition zones for all active β-lactams, including representatives of the penicillin, cephalosporin, and carbapenem classes (Table 1, Strain #3279 and #3322). Determination of the MIC of a subset of β-lactams (Table 2) showed that introducing the gene encoding Pbp2c from either *C. jeikeium* or *C. urealitycum* into *C. glutamicum* increased the MIC to levels equivalent to those observed for resistant strains of *C. jeikeium* (CjkR or K411) or *C. urealyticum* (DSM7109). A similar set of experiments was also performed using the E-test® system. In solid medium, the MIC were slightly different and resistance was underestimated (Supplementary Table S4).

**Table 2:** Sensitivity of *Corynebacterium* strains determined by the microdilution assay.

	MIC (μg/mL) for strains^a^							
	*C. jeikeium*			*C. urea*^b^	*C. glutamicum*^c^			
Antibiotic	CjkS [none]	CjkR [*jk0412*]	K411 [*jk0412*]	CuR [*Cu1571*]	RES167 [none]	#3278 [none]	#3279 [*jk0412*]	#3322 [*Cu1571*]
Benzylpenicillin	2	1,024	1,024	>1,024	0.5(1)	0.5(1)	>1,024	>1,024
Benzylpenicillin/clavulanate^d^	2	1,024	1,024	>1,024	0.25(0.5)	0.25(0.5)	>1,024	>1,024
Meropenem	1	16 (64)	128 (256)	>1,024	0.25	0.25	>1,024	>1,024
Meropenem/clavulanate^d^	1	512	512	>1,024	<0.0625	<0.0625	>1,024	>1,024
Clavulanate	>1,024	>1,024	>1,024	>1,024	16	16	>1,024	>1,024

The MICs were determined after 24 h of incubation. If an increase in the median (≥2-fold) of the MICs was detected after an additional incubation of 24 h, the median of the MICs observed at 48 h are indicated in brackets. Concentrations used for CMI determination were from 1,024 to 0.0625 μg/mL. ^a^Values are the median of at least three determinations. ^b^*C. urea* = *C. urealyticum*. ^c^*C. glutamicum* *#3278, #3279, and #3322* are derivatives of strain RES167 harboring plasmid pMM36, pMM36ΩP*_trc_-jk0412* (pML2), and pMM36ΩP*_trc_-cu1571*, respectively. ^d^Clavulanate 10 μg/mL.

**Table 3:** Sensitivity of *Corynebacterium* strains determined by the E-test® assay.

	Benzylpenicillin			Amoxicillin		
		CA-SFM	EUCAST		CA-SFM	EUCAST
Strain	MIC^a^ (μg/mL)	V1-2023 (0.125–0.125)	V14.0 2024 (0.001–1)	MIC^a^ (μg/mL)	V1-2023 (2–8)	V14.0 2024 –
*C. jeikeium*						
CIP82.51 (CjkS)	1.5	R	R	1.5	S	NA
CIP103337 (CjkR)	>256	R	R	>32	R	NA
K411	>256	R	R	>32	R	NA
*C. urealyticum*						
DSM7109 (CuR)	>256	R	R	>32	R	NA
*C. glutamicum*						
RES167	0.19	R	F	0.25	S	NA
#3278^b^	0.19	R	F	0.25	S	NA
#3279^b^	4+Sq^c^	R	R	4 + Sq	R	NA
#3322^b^	4 + Sq	R	R	4 + Sq	R	NA

Interpretation of the benzylpenicillin and amoxicillin MICs determined by the E-test® assay after 48 h of incubation. NA: not applicable, no breakpoint. F: sensitive at high dosage. ^a^Values are the median of at least three determinations. ^b^*C. glutamicum* *#3278, #3279, and #3322* are derivatives of strain RES167 harboring plasmid pMM36, pMM36Ω*P_trc_*-*jk0412* (pML2), and pMM36Ω*P_trc_*-*cu1571*, respectively. ^c^Sq, presence of scatter colonies in the inhibition zone.

Overall, these results showed that expression of the *pbp2c* genes from *C. jeikeium* and *C. urealyticum* confered a broad-spectrum resistance to β-lactams in a sensitive host.

### Induction of β-lactam resistance by clavulanate and ampicillin in *Corynebacterium jeikeium*

**Figure 1:**
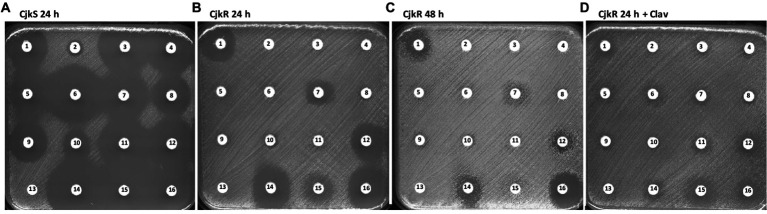
Antibiogram by the disk diffusion assay performed on MH-F agar for **(A)** *Corynebacterium jeikeium* strains CIP82.51 (CjkS), **(B)** CIP103337 (CjkR) incubated 24 h, **(C)** CIP103337 (CjkR) incubated 48 h, and **(D)** CIP103337 (CjkR) cultivated in the presence of clavulanate (Clav) at 10 μg/mL and incubated 24 h. Disks contain **1**: cefoxitin (30 μg), **2**: benzylpenicillin (1 U), **3**: ticarcillin (75 μg), **4**: ampicillin (10 μg), **5**: cefalexin (30 μg), **6**: amoxicillin/clavulanate (20 μg/ 10 μg), **7**: ceftaroline (5 μg), **8**: piperacillin (30 μg), **9**: piperacillin/tazobactam (30 μg/6 μg), **10**: cefotaxime (5 μg), **11**: ticarcillin/clavulanate (75 μg/10 μg), **12**: cefepime (30 μg), **13**: clavulanate (10 μg), **14**: meropenem (10 μg), **15**: imipenem (10 μg), or **16**: ertapenem (10 μg).

**Figure 2:**
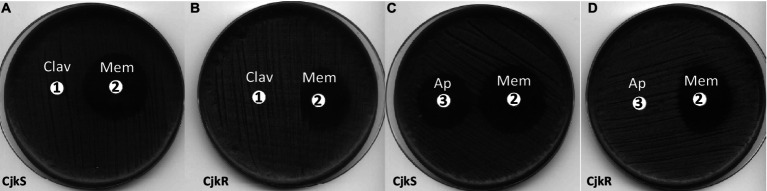
Double disk diffusion assay for *C. jeikeium* strains CIP82.51 (CjkS) **(A,C)** and CIP103337 (CjkR) **(B,D)**. Plates were incubated for 24 h on MH-F agar. Disk **1**: clavulanate (Clav, 10 μg), disk **2**: meropenem (Mem, 10 μg), disk **3**: ampicillin (Ap, 10 μg).

A wide range of β-lactam antibiotics have been tested in order to study the activity of the different classes and to determine if the resistance mechanism could be inducible (Table 1; Figure 1). Comparison of the antibiograms of *C. jeikeium* CIP82.51 (CjkS) (Figure 1A) and *C. jeikeium* CIP103337 (CjkR) (Figures 1B,C) showed that inhibition zones persisted despite the presence of the *pbp2c* gene in the resistant strain around disks containing cefoxitin (disk 1, 30 μg), ceftaroline (disk 7, 5 μg), cefepime (disk 12, 30 μg), meropenem (disk 14, 10 μg), and ertapenem (disk 16, 10 μg). We observed antagonism between clavulanate (disk 13, 10 μg) and meropenem (disk 14, 10 μg). We observed such antagonism in the double disk diffusion assay, which showed the typical “D” shape blunting of the inhibition zone around the disk containing meropenem in the vicinity of disks containing clavulanate (Figures 2A,B) or ampicillin (Figures 2C,D). This observation suggested that clavulanate induced resistance to meropenem. Thus, we performed an antibiogram of *C. jeikeium* CIP103337 (CjkR) in a clavulanate-containing medium (Figure 1D). In the presence of clavulanate, the strain grew in contact with all β-lactam containing disks, suggesting that cefoxitin (disk 1, 30 μg), ceftaroline (disk 7, 5 μg), cefepime (disk 12, 30 μg), meropenem (disk 14, 10 μg), and ertapenem (disk 16, 10 μg) remained partially active against the CjkR strain because they were unable to completely induce β-lactam resistance in 24 h (Figure 1B) and even 48 h for ertapenem (Figure 1C). Thus, a 48 h incubation period was necessary for a correct interpretation of antibiograms and evaluation of resistance to ceftaroline, cefepime, meropenem, and imipenem. For ertapenem, a 48 h incubation was not sufficient and the induction with clavulanate in the media was needed to correctly detect resistant strains.

**Figure 3:**
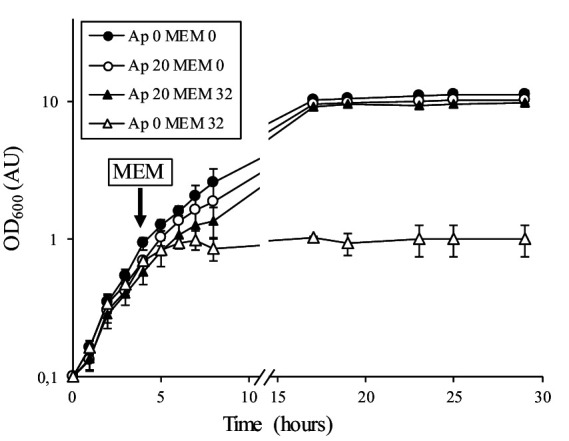
Growth curves of *C. jeikeium* K411 in the absence or presence of meropenem. An overnight culture of strain K411 diluted to 0.1 OD_600_ was grown in the absence (Ap 0) or presence (Ap 20) of ampicillin at 20 μg/mL for 4 h (OD_600_ of approximately 0.4). Meropenem (32 μg/mL, MEM 32) was added to the indicated cultures. OD_600_ values are the mean ± standard error from three determinations.

To distinguish between inducible resistance, which requires a 24 h prolonged incubation, and standard bacterial growth under subinhibitory antibiotic conditions, we performed experiments in the presence of high inhibitory concentrations of meropenem (32 and 128 μg/mL). We also tested the induction of β-lactam resistance by following growth in the presence or absence of an inducer (Figure 3). We used ampicillin as the inducer because this antibiotic produced the same “D-shape” blunting of the inhibition zone of meropenem as clavulanate does (Figure 2C). From an overnight culture diluted to 0.1 OD_600_ in fresh medium containing no inducer, the addition of meropenem (32 μg/mL) after 4 h of incubation inhibited growth of *C. jeikeium* K411 (Figure 3, white triangle). In contrast, prior growth of the strain in the presence of a sub-inhibitory concentration of ampicillin (20 μg/mL) for 4 h completely prevented inhibition by meropenem (Figure 3, black triangle).

**Figure 4:**
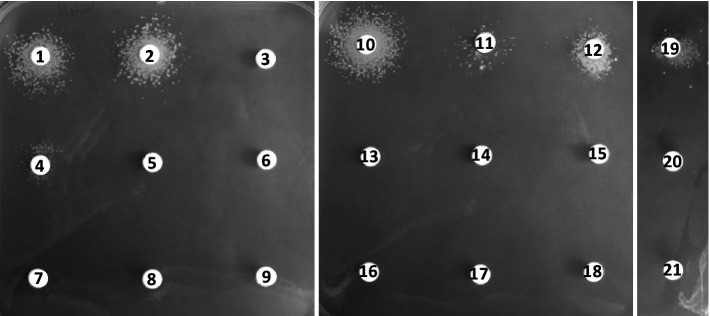
Induction of meropenem resistance by the disk diffusion assay for strain *C. jeikeium* K411. Plates were incubated for 48 h. The assays were performed on BHI agar containing 0.5% Tween 80 and meropenem at 128 μg/mL. The disks contained **1**: ampicillin (10 μg), **2:** oxacillin (5 μg), **3:** piperacillin (30 μg), **4:** cephalothin (30 μg), **5:** cefoxitin (30 μg), **6:** ceftazidime (30 μg), **7:** cefepime (30 μg), **8:** colistin (30 μg), **9:** chloramphenicol (30 μg), **10:** benzylpenicillin (10 U), **11:** amoxicillin (25 μg), **12:** amoxicillin/clavulanate (20 μg/10 μg), **13:** gentamicin (10 μg), **14:** erythromycin (15 μg), **15:** norfloxacin (10 μg), **16:** vancomycin (5 μg), **17:** teicoplanin (30 μg), **18:** tetracycline (30 μg), **19:** imipenem (10 μg), **20:** meropenem (10 μg), or **21:** ertapenem (10 μg).

We tested induction of the resistance profile by various β-lactam and non-β-lactam antibiotics when performing experiments during which a high inhibitory concentration of meropenem (128 μg/mL) was added to the agar plate medium and the potential inducers loaded onto the disk (Figure 4). In these experiments, inducible resistance was mediated by most β-lactams, not by non-β-lactam antibiotics. Ampicillin (disk 1, 10 μg), oxacillin (disk 2, 5 μg), benzylpenicillin (disk 10, 10 U), amoxicillin/clavulanate (disk 12, 20/10 μg), and imipenem (disk 19, 10 μg) were strong inducers of meropenem resistance, whereas cephalothin (disk 4, 30 μg) and amoxicillin alone (disk 11, 25 μg) appeared to be weaker inducers.

### Induction of Pbp2c production by clavulanate and ampicillin

**Figure 5:**
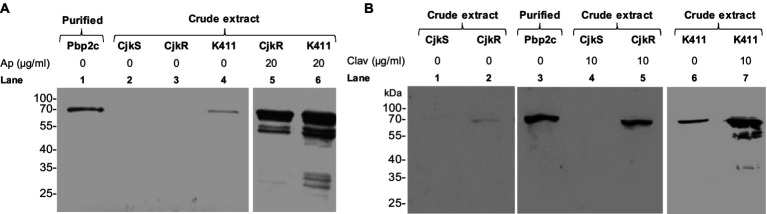
Impact of ampicillin **(A)** or clavulanate **(B)** on the level of Pbp2c production. *C. jeikeium* strains were grown for 24 h in the presence of ampicillin (Ap) or clavulanate (Clav). Crude extracts (10 μg) were analyzed by Western blotting with polyclonal antibodies raised against the Pbp2c protein encoded by *jk0412*. The extracts were prepared from strain *C. jeikeium* CIP82.51 (CjkS), a β-lactam-sensitive strain that does not harbor *pbp2c*, and from the β-lactam resistant strains CIP103337 (CjkR) and K411. Purified Pbp2c (3 ng) was used as a control.

We used immunodetection of Pbp2c to determine whether the induction of β-lactam resistance by clavulanate and ampicillin resulted from increased synthesis of Pbp2c in response to these drugs. Bacteria were cultivated under agitation during 24 h in liquid media in the absence or the presence of antibiotics. A polyclonal anti-Pbp2c mouse antiserum was used for Western blot analyses of *C. jeikeium* protein extracts and revealed a protein band of *ca.* 63 kDa for *C. jeikeium* K411 and CIP103337 (CjkR) whith the *pbp2c* gene (Figure 5). This protein band migrated at an equivalent position as did the purified Pbp2c and was not detectable in protein extract from *C. jeikeium* CIP82.51 (CjkS) which lacks *pbp2c*. These results indicated that the mouse antiserum was specific to Pbp2c. The intensity of the signal corresponding to the Pbp2c protein was strongly enhanced in the protein extracts from cultures performed in the presence of ampicillin (Figure 5A) or clavulanate (Figure 5B) but barely detectable in the absence of these drugs.

### Inactivation of Pbp2c by the formation of adducts with β-lactams

**Figure 6:**
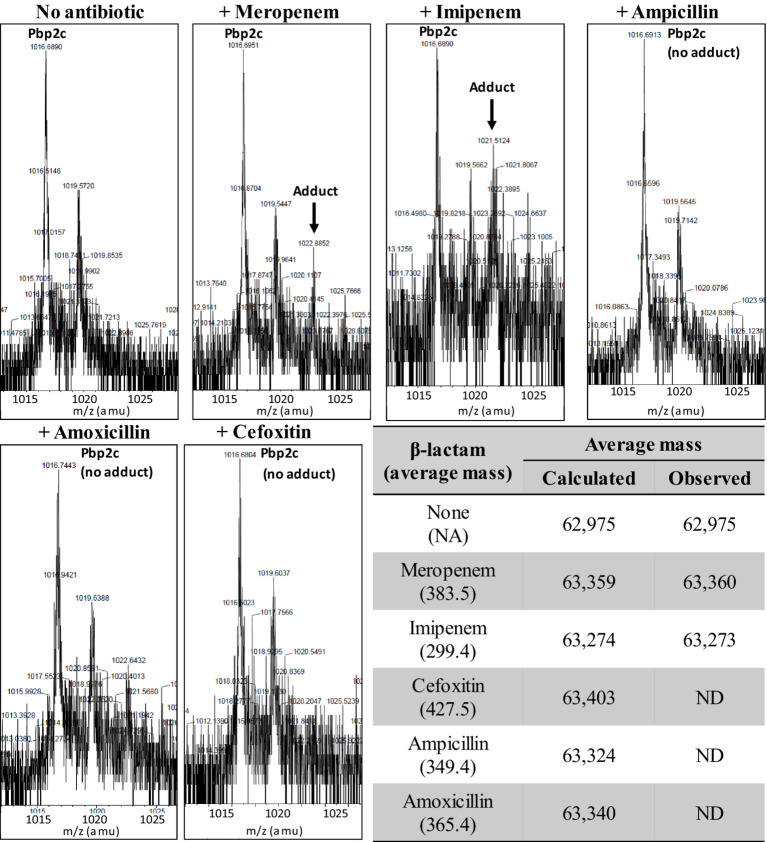
Formation of adducts between Pbp2c and β-lactams. Pbp2c was incubated without or with antibiotics. Peaks at m/z 1,016.7 and 1,019.6 correspond to the [M + 62H]^62+^ of the native protein (deduced mass average of 62,975 Da) and a spontaneous α-*N*-6-phosphogluconoylation of the poly histidine tag (Geoghegan et al., 1999). ND: not detected.

We explored the inactivation of Pbp2c by β-lactams by investigating the formation of adducts in the presence of various β-lactams. Binding of the drugs to the enzyme was tested by electrospray mass spectrometry, as previously described (Mainardi et al., 2007). In these experiments (Figure 6), we detected no adducts between Pbp2c and ampicillin, cefoxitin, or amoxicillin. Incubation of Pbp2c with imipenem allowed the detection of adducts with an average mass matching the addition of the mass of imipenem, but at a low level relative to the signal of the free protein. Adducts matching increments of the average mass of meropenem were also detected but only at a very low level.

### β-Lactamase activity

In order to investigate how the level of β-lactam resistance was the result of the presence of an unidentified β-lactamase or not, we tested the presence of a β-lactamase activity on bacterial extracts from *C. jeikeium* K411 and CjkR, cultivated with or without β-lactam (amoxicillin and/or clavulanate), from CjkS, from the *C. glutamicum* RES167 strains transformed with either pML1, pML2, pML3, in the purified Pbp2c protein extract, and from *M. chelonae* as a positive control. With the exception of the *M. chelonae* positive control, no β-lactam hydrolysis was detected, indicating that the observed resistance was due to the overproduction of a low-affinity Pbp and not from hydrolysis of the β-lactam antibiotics.

### Distribution of the Pbp2c-encoding gene among clinical isolates of *Corynebacteriaceae*

We then investigated the presence of the gene coding for Pbp2c in medical settings. The gene has been detected by PCR using the primer pair Pbp2c_PCR_Fw and Pbp2c_PCR_Rev (Supplementary Table S2), which yielded a 1,401-bp fragment (Supplementary Figure S2) containing the SxxK and SxN catalytic domains of Pbp2c and the positions corresponding to V361 and S372, which were found to be substituted in CjkR-4, a highly resistant strain derived from CjkR against ampicillin (Lavollay et al., 2009). We systematically found the *pbp2c* gene in strains with a β-lactam-resistance phenotype, whereas it was absent from sensitive isolates of *C. jeikeium* or other *Corynebacteria* species (Table 4).

**Table 4:** PCR detection of the *pbp2c* gene in various isolates.

Isolate	Characteristics	Origin	PCR *pbp2c*
*Corynebacterium jeikeium*			
K411	Reference strain, β-lactam-resistant^a^, nucleotide accession number NC_007164	NCTC (Tauch et al., 2005)	+
CIP82.51(CjkS)	Reference strain, β-lactam sensitive^b^	CIP	−
CIP103337 (CjkR)	Reference strain, β-lactam resistant	CIP	+
B44229	Clinical isolate, β-lactam-resistant	HS (Riegel et al., 1994)	+
B75089	Clinical isolate, β-lactam-resistant	HS (Riegel et al., 1994)	+
#2017-1A2	Clinical isolate, β-lactam-sensitive	IMM	−
*Corynebacterium urealyticum*			
DSM7109 (CuR)	Reference strain, β-lactam resistant, nucleotide accession number NC_010545	CIP (Tauch et al., 2008)	+
ATCC 43043	Reference strain, β-lactam resistant	ATCC	+
A12818	Clinical isolate, β-lactam-resistant	HS	+
A25965	Clinical isolate, β-lactam-resistant	HS	+
*Corynebacterium amycolatum*			
#2921	Clinical isolate, β-lactam-resistant	HEGP	+
#2922	Clinical isolate, β-lactam-sensitive	HEGP	−
#2926	Clinical isolate, β-lactam-resistant	HEGP	+
#2928	Clinical isolate, β-lactam-sensitive	HEGP	−
#2929	Clinical isolate, β-lactam-resistant	HEGP	+
#2023-6E7	Clinical isolate, β-lactam-resistant	IMM	+
*Corynebacterium tuberculostearicum*			
#3057	Clinical isolate, β-lactam-resistant	HEGP	+
*Corynebacterium striatum*			
#3635	Clinical isolate, β-lactam-resistant	HEGP	+
#3637	Clinical isolate, β-lactam-sensitive	HEGP	−
#2023-ID6	Clinical isolate, β-lactam-resistant	IMM	+
#2023-1H6	Clinical isolate, β-lactam-resistant	IMM	+
#2023-2A3	Clinical isolate, β-lactam-resistant	IMM	+
#2023-2H3	Clinical isolate, β-lactam-sensitive	IMM	−
#2023-4F2	Clinical isolate, β-lactam-sensitive	IMM	−
#2023-6C6	Clinical isolate, β-lactam-resistant	IMM	+
*Corynebacterium propinquum*			
#3639	Clinical isolate, β-lactam-sensitive	HEGP	−
*Corynebacterium aurimucosum*			
#2023-1F5	Clinical isolate, β-lactam-sensitive	IMM	−

^a^β-Lactam resistant: ampicillin MIC >2 μg/mL (CA-SFM V. 2.02019). ^b^β-lactam sensitive: ampicillin MIC ≤2 μg/mL (CA-SFM V. 2.02019). NCTC, National Collection of Type Cultures; CIP, Collection Institut Pasteur; HS, Hôpitaux Universitaires de Strasbourg, France; ATCC, American Type Culture Collection; HEGP, Hôpital Européen Georges Pompidou, France; AP, Hôpital Ambroise Paré, France; IMM, Institut Mutualiste Montsouris, France.

### Structure of the *pbp2c* locus

**Figure 7:**
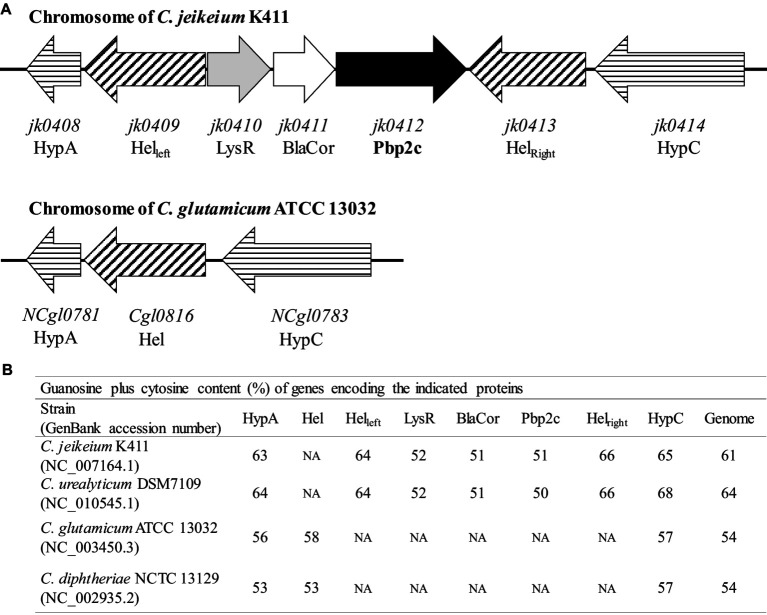
Schematic organization and G + C content of the *pbp2c* locus. **(A)** Map of the *pbp2c* locus in *C. jeikeium* K411 and comparison with the homologous region of the *C. glutamicum* ATCC 13032 locus. **(B)** G + C content of relevant genes and comparison with that of whole genome.

In order to understand the origin of the *pbp2c* gene, we carried out *in silico* genome analyses of *Corynebacteriaceae* species with *jk0412* gene analogs. Sequence comparisons and analyses showed that the *pbp2c* gene found in *Corynebacteriaceae* was always associated with the presence of two additional genes, *jk0410* and *jk0411*, namely, not detected in the *pbp2c*-negative strains. These two genes, located upstream of *jk0412* and transcribed in the same orientation (Figure 7A), have the same G/C content as *jk0412*. This content was found to be 10% lower than the G/C content of the host strains genomes (Figure 7B). This substantial divergence in the G/C content suggests a common and exogenous origin for the locus containing these three genes (referred here as to the *pbp2c* locus).

### The *jk0410* and *jk0411* genes are involved in the inducible regulation of Pbp2c production

**Figure 8:**
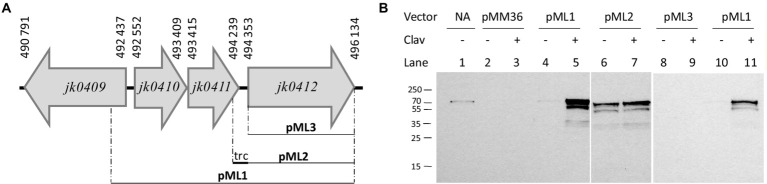
**(A)** Schematic representation of the constructs pML1, pML2, and pLM3. **(B)** Western blot of total proteins extracted from *C. glutamicum* RES167 transformed with pML1, pML2, or pML3. Strains were cultivated overnight without antibiotics, diluted to an OD_600_ of 0.1 in fresh BHI media complemented (+) or not (−) with clavulanate (clav, 1 μg/mL), and cultivated under agitation for 24 h. Protein extracts (10 μg) were analyzed by Western blotting with polyclonal antibodies raised against the Pbp2c protein encoded by *jk0412.* Line 1: purified Pbp2C; Line 2–3: RES167 harboring pMM36 (#3278); Line 4–5 and 10–11: RES167 harboring *pMM36Ωjk0410-jk0411-jk0412* (#3815); Line 6–7: RES167 harboring pMM36Ω*P_trc_-jk0412* (#3279); Line 8–9: RES167 harboring pMM36Ω*jk0412* (#3812).

Similarity analyses of the *jk0410* and *jk0411* gene products using the protein BLAST program disclosed homology with regulatory proteins of the transcriptional regulator LysR family and the class A β-lactamase family, respectively. We explored the role of the conserved *jk0410* and *jk0411* genes in the regulation of Pbp2c expression by producing additional constructs (Figure 8A). The first construct, pML1, contained the complete gene set (*jk0410*, *jk0411*, and *jk0412*) incorporated into pMM36. The pMM36 plasmid containing the gene encoding Pbp2c (*jk0412*) under the control of the exogenous *P_trc_* promoter was designated pML2. Finally, the pMM36 plasmid construct containing the *pbp2c* gene alone (*jk0412*), without the *P_trc_* promoter, was designated pML3. Each of the three plasmids pML1, pML2, and pML3, were introduced into *C. glutamicum* RES167. *C. glutamicum* strains containing either pML1, pML2 or pML3 were cultivated overnight in 10 mL of medium without inducer. Each culture was diluted in fresh culture medium until DO_600_ reached 0.1 and was cultivated for 24 h in the presence or absence of the inducer (clavulanate, 1 μg/mL). Total proteins were extracted and a Western blot experiment was performed to detect the expression of the *jk0412* gene using anti-Pbp2c antibodies (Figure 8B). In the presence of clavulanate, Pbp2c was detected only when bacteria were transformed with pML1(the complete *jk0410*, *jk0411*, *jk0412* gene cluster). The Pbp2c protein was not detected in the absence of the *jk0410* and *jk0411* genes or without the *P_trc_* promoter (pML3), independently of whether the bacteria were grown in the presence or absence of clavulanate. Last, the Pbp2c production was restored by introducing the *pb2c* gene under the control of the *P_trc_* promoter, in the presence or absence of clavulanate in the medium.

### Putative roles of the proteins encoded by the *jk0410* and *jk0411* genes

The conserved motives SxxK, SDx and KTG found in β-lactamases were systematically searched in proteins encoded by the *C. jeikeium K411* genome. Twelve genes were identified that could encode proteins with β-lactamase/transpeptidase-like domains (IPR012338). Seven of these genes encode previously identified proteins: Pbp1a (*jk2069*), Pbp1b (*jk1977*), Pbp2a (*jk0039*), Pbp2b (*jk1160*), Pbp2c (*jk0412*), FtsI (*jk0744*), and Pbp4 (*jk075*) (Lavollay et al., 2009). Out of the five other genes, namely *jk0411*, *jk0584*, *jk0658*, *jk1553* and *jk2026*, only *jk0411* encodes a protein with all the three conserved β-lactamase/transpeptidase-like motives (Supplementary Figure S3).

The *jk0411* gene encodes a 276 amino acid protein, hereafter referred to as Bla_Cor_. The amino acid sequence of Bla_Cor_ display the three amino acid motifs SxxK at positions 45 to 48, SDN at positions 106 to 108, and KTG at positions 220 to 222. The sequence of was also compared with the constitutive β-lactamase BlaC from *M. tuberculosis* (Supplementary Figure S3). Although the structural alignment (RMS of 9.5 Å) of the AlpahaFold models for Bla_Cor_ (AF-Q4JX89) and BlaC (PDB code 6 N14) seemingly indicate that the three conserved domains are positioned in Bla_Cor_ as in BlaC (data not shown), Bla_Cor_ is 39 amino acids shorter than BlaC, an organization that could contribute to the inability of Bla_Cor_ to hydrolyze β-lactams. To assess the ability of Bla_Cor_ to hydrolyze β-lactams, the protein in fusion with a 6His tag at the *N*-terminus position of Bla_Cor_ was purified from crude extracts of *E. coli* expressing *jk0411*. Size-exclusion chromatography and mass spectroscopy indicated that Bla_Cor_ was purified as a monomer. Incubation of purified Bla_Cor_ with clavulanate resulted in the formation of a covalent +155 Da trans-enamine adduct (Hugonnet and Blanchard, 2007) which was detected by mass spectrometry (Supplementary Figure S4). A Ser to Ala substitution at position 45 of Bla_Cor_, corresponding to the catalytic Ser residue in Pbp and β-lactamases, abolished the binding of β-lactams to Bla_Cor_ (Supplementary Figure S4) indicating that Bla_Cor_ is a *bona fide* Pbp. The incubation of Bla_Cor_ with ampicillin resulted in the formation of a covalent +349 Da adduct which was also detected by mass spectrometry (Supplementary Figure S4). To determine whether Bla_Cor_ could act as a β-lactamase, the protein was incubated with the chromogenic cephalosporin nitrocefin. Even if the nitrocefin-Bla_Cor_ adduct was detected by mass spectrometry (data not shown), incubation with nitrocefin (100 μM) with several concentrations of Bla_Cor_ (0, 5, 10, 15, and 20 μM) did not result in nitrocefin hydrolysis. All together these results indicate the absence of β-lactamase activity for this enzyme.

## Discussion

Considerable variations in the sensitivity of *Corynebacteriaceae* species and strains against β-lactams were reported (Soriano et al., 1995). Several species were experimentally shown to be sensitive to penicillin and ampicillin (e.g., *C. diphteriae*, *Corynebacterium pseudotuberculosis*, *Corynebacterium ulcerans*, and *Corynebacterium pseudodiphteriticum*) (Funke et al., 1997), whereas others have shown low sensitivity to β-lactams. Resistance mechanisms were almost unknown at the beginning of this study (Soriano et al., 1995; Funke et al., 1996; Weiss et al., 1996; Funke et al., 1997).

The *pbp2c* gene was previously found to be present in the resistant *C. jeikeium* CIP103337 strain genome (CjkR) but absent in the sensitive *C. jeikeium* CIP82.51 strain genome (CjkS), suggesting that Pbp2c could be one determinant of the β-lactam resistance observed in *C. jeikeium* CIP103337 (Lavollay et al., 2009). Here, we showed that the heterologous expression of the Pbp2c-encoding-gene from *C. jeikeium* CIP103337 in *C. glutamicum* RES167 confered a β-lactam resistance to this sensitive species and strengthen the role of Pbp2c as a potent, key determinant, of β-lactam resistance in multidrug-resistant *C. jeikeium* and *C. urealyticum*. We found that the *jk0412* gene, encoding Pbp2c, was present in the genomes of β-lactam-resistant *Corynebacterium* species, such as *C. amycolatum*, *Corynebacterium tuberculostearicum*, and *Corynebacterium striatum*, (Table 4) and was recently shown to be present in a mobile element in *C. diphtheriae* (Hennart et al., 2020). The nucleotide sequence of *pbp2c* was found to be highly conserved, with an identity value ≥99% in multidrug-resistant *Corynebacteriaceae* species (Hennart et al., 2020). These results highlight the use of a *pbp2c*-based screening method as a powerful diagnostic tool to improve the detection of infections and the management of patients infected with *Corynebacteriaceae*.

We tested the induction of β-lactam resistance using several *C. glutamicum* strains: one carrying the *pbp2c* gene (*jk0412*) alone, one with the gene under the control of the P*_trc_* promoter, and one in combination with the *jk0410* and *jk0411* genes (complete *pbp2c* locus). We showed that *jk0412* is one of the main gene involved in high levels of β-lactam resistance in *Corynebacteriaceae*. We also showed that Pbp2c expression was under the control of the *jk0410* and/or *jk411* genes which encode for a LysR analog, likely a transcriptional regulator and the β-lactamase analogue (Bla_Cor_), respectively. We showed that Bla_Cor_ was able to covalently link β-lactams, but was unable to hydrolyze these compounds. These results suggested that the formation of stable, covalent bonds between Bla_Cor_ and β-lactam products could play a role in Pbp2c expression by a mechanism yet to be discovered. We found that the induction was modulated by the β-lactam used. Ampicillin, oxacillin, benzylpenicillin, and imipenem were strong inducers, whereas cefoxitin, ceftaroline, cefepime, meropenem, and ertapenem were weaker inducers. We also found that clavulanate, a β-lactamase inhibitor of the β-lactam family but without antibacterial activity, was also a strong inducer. These differences in resistance induction raised concerns about the protocol for the correct detection of resistant strains of *Corynebacteriaceae*. In clinical practice, current EUCAST (EUCAST Clinical Breakpoint Tables v. 14.0, 2024)^1^ and CA-SFM (V1.0 2023) guidelines recommend testing *Corynebacteriaceae* using benzylpenicillin with a breakpoint at 1 μg/mL and 0.125 μg/mL, respectively (Table 3). As the MIC of this molecule for sensitive strains is 2 μg/mL (Table 2), some strains from the *Corynebacteriaceae* family could be misclassified as resistant to all β-lactam antibiotics.

We also observed that testing sensitivity to ertapenem or meropenem using a classical 24 or 48 h incubation protocol also failed to reliably detect *pbp2c*-mediated resistant strains because the induction phenomenon occurred too slowly to be detected under these conditions. A straightforward, rapid, and easily implementable procedure in medical laboratory environments can overcome such drawbacks. This procedure, based on the E-Test strip superimposition method, described for the study of sensitivity to the ceftazidime-avibactam-aztreonam association (Davido et al., 2017), requires pre-incubation of an agar plate for 10 min with an E-Test that contains a powerful inducer (e.g., an amoxicillin-clavulanate association), the removal of the first strip, and its substitution with a new strip containing the carbapenem of interest (ertapenem or meropenem). The result of this E-test revealed the true sensitivity of the strain, whether or not it possesses the gene encoding the Pbp2c protein (Supplementary Figure S5).

In conclusion, this study provides an improved understanding of the mechanism of β-lactam resistance in *Corynebacteriaceae*. We showed that resistance was mediated by a low affinity Pbp protein (Pbp2c) and not by the acquisition of a β-lactamase-encoding gene. Detection of *Corynebacteriaceae* strains harboring the *pbp2c* gene could therefore contribute to improve the management of patients with complex infections implying *Corynebacteriaceae*. To highly notice, this would make possible avoiding the use of second-line antibiotics, which can expose patients to side effects, such as renal failure with vancomycin (Lu et al., 2023), or the emergence of highly resistant strains with daptomycin (McElvania TeKippe et al., 2014; Mitchell et al., 2021).

## Data availability statement

The raw data supporting the conclusions of this article will be made available by the authors, without undue reservation.

## Ethics statement

The animal study was approved by institutional and national ethical guidelines (Agreement n°783223; approved by the French Ministry of Higher Education and Research with APAFIS#11465-2016111417574906v4). The study was conducted in accordance with the local legislation and institutional requirements.

## Author contributions

ML: Conceptualization, Formal analysis, Investigation, Methodology, Resources, Supervision, Validation, Writing – original draft, Writing – review & editing. CB: Investigation, Methodology, Writing – original draft. VM: Writing – original draft, Investigation. FC: Writing – review & editing. AG: Resources, Writing – review & editing. MF: Writing – review & editing, Writing – original draft, Validation, Methodology, Investigation, Formal analysis.

## Footnotes

^1^https://www.eucast.org
