# Supplementary material for: Exploration of the role of the penicillin binding protein 2c (Pbp2c) in inducible β-lactam resistance in Corynebacteriaceae
Source: Front Microbiol. 2024 May 9;15:1327723. doi: 10.3389/fmicb.2024.1327723 (PMC11111852; doi:10.3389/fmicb.2024.1327723)
Supplement: Supplementary file 1 [file Data_Sheet_1.pdf]

SUPPLEMENTARY INFORMATION

Exploration of the role of the penicillin binding protein 2c (Pbp2c) in inducible β-lactam resistance in *Corynebacteriaceae.*

Marie Lavollay[1,2,3*], Céline Buon[1], Vincent Le Moigne[4], Fabrice Compain[3], Armel Guyonvarch[5], and Matthieu Fonvielle[5*]

Table S1. Origin and characteristics of bacterial strains

Strain	Characteristics	Reference
Escherichia coli		
Top 10	F- *mcrA* Δ(*mrr-hsd*RMS-*mcr*BC) Φ80*lac*ZΔM15 Δ*lac*X74 *rec*A1 *ara*D139 Δ(*araleu*)7697 *gal*U *gal*K *rps*L (StrR) *end*A1 *nup*G	Invitrogen
BL21 (DE3)	*fhuA2 [lon] ompT gal (λ DE3) [dcm] ΔhsdS λ DE3 = λ sBamHIo ΔEcoRI-B int::(lacI::PlacUV5::T7 gene1) i21 Δnin5*	Invitrogen
Corynebacterium jeikeium		
K411	Reference strain, β-lactam-resistant[a], nucleotide accession number NC_007164	NCTC (1)
CIP82.51(CjkS)	Reference strain, β-lactam sensitive[b]	CIP
CIP103337 (CjkR)	Reference strain, β-lactam resistant	CIP
Corynebacterium urealyticum		
DSM7109 (CuR)	Reference strain, β-lactam resistant, nucleotide accession number NC_010545	CIP (2)
ATCC 43043	Reference strain, β-lactam resistant	ATCC
Corynebacterium glutamicum		
ATCC13032	Reference strain, accession number NC_003450	ATCC
RES167	Restriction deficient mutant of ATCC13032	(3)
#3278	RES 167 harboring vector pMM36	This study
#3279	RES 167 harboring pMM36ΩP_{trc}-*jk0412*	This study
#3322	RES 167 harboring pMM36Ω*ptrc*Ω*cu1571*	This study
#3812	RES 167 harboring pMM36Ω*jk0412*	This study
#3815	RES 167 harboring pMM36Ω*jk0410-jk0411-jk0412*	This study
Mycobacterium chelonae		
#3635	Clinical isolate, β-lactamase producer	AP

[a]β-lactam resistant: Ampicillin MIC >2 µg/mL (CASFM 2013), [b]β-lactam sensitive: ampicillin MIC ≤ 2µg/mL (CASFM 2013). Abbreviations: NCTC, National Collection of Type Cultures; CIP, Collection Institut Pasteur ATCC, American Type Culture Collection; P_{trc}, *trc* promotor ; AP, Hôpital Ambroise Paré, France.

Table S2. Sequence of primers used in this study

Oligonucleotide	Sequence (5' →3')	Purpose
Fwjk0412_1-20L	ATCCATGGTGACTAAGCACAATCGTTTC	Expression (E)
Fwjk0412_93-113S	ATCATATGGAATCAGACTCAGACGTATT	Purification (P)
Revjk0412_1782-1762	ATCTCGAGTTATTGAATTCCAGAGAATTTC	E+P
Ptrc_Fw_4980	TTCATACACGGTGCCTGACTGC	Expression
Ptrc_Rev_353	AATCTCGAGCTCGAATTCAGCTGC	Expression
jk0412R1782-1759	TTGTCGACCTCGAGTTATTGAATTCCAGAGAAT TTC TG	Expression
jk0411F769-788	TTGGATCCATGAGTACTCACGATGGTGC	Expression
Pbp2c_PCR_Fw	TTGGTCCAAAGATGGTGTGGAGTTGGAGAGCAATGGTCGGC	*pbp2c* detection
Pbp2c_PCR_Rev	GCCAATTCCCCTGTCGTTCCAGCGAACCAGCCATTGGAAAG	*pbp2c* detection
jk0411_Fw_XbaI	ATTCTAGATGACTGCACGGATGGAGAT	Expression
jk0411_Rev_NotI	ATGCGGCCGCTAACGAAGAGCGTCCACGA	Expression

Table S3. Origin and characteristics of the plasmids

Plasmid	Characteristics	Reference
pCR™-Blunt	Cloning of blunt-end PCR products, Km^r	Invitrogen
pKK388-1	*E. coli* expression vector containing the *trc* promoter (P_{trc}), Ap^r	(4)
pET2818	*E. coli* expression vector, Ap^r	Novagen
pET-TEV	Derivative of expression vector pET28a for production of fusion proteins containing 6 x His Tag and a TEV protease cleavage site instead of a thrombin cleavage site, Km^r	(5)
pMM36	*E. coli* - *C. glutamicum* shuttle vector containing the *trpA* terminator; Cm^r	(6)
pBluntΩP_{trc}	P_{trc} inserted into pBlunt	This study
pBluntΩ*cu1571*	*cu1571* inserted into pBlunt	This study
pET2818Ω*cu1571*	*cu1571* inserted into pET2818	This study
pET2818ΩP_{trc}-*cu1571*	P_{trc} and *cu1571* inserted into pET2818	This study
pMM36ΩP_{trc}-*cu1571*	*cu1571* inserted into pMM36	This study
pBluntΩ*jk0412*	*jk0412* inserted into pBlunt	This study
pET-TEVΩ*jk0412*	*jk0412* inserted into pET-TEV	This study
pET2818Ω*jk0412*	*jk0412* inserted into pET2818	This study
pET2818ΩP_{trc}-*jk0412*	$P_{trc}$and*jk0412* inserted into pET2818	This study
pMM36Ω*jk0410-jk0411-jk0412* (pML1)	*jk0410, jk0411 and jk0412* inserted into pMM36	This study
pMM36ΩP_{trc}-*jk0412* (pML2)	P_{trc} and *jk0412* inserted into pMM36	This study
pMM36Ω*jk0412* (pML3)	jk0412 inserted into pMM36	This study

Table S4. Sensitivity of *Corynebacterium* strains determined by the E-test® assay

Antibiotic	MIC (µg/mL) for strains[a]							
	C. jeikeium			*C. urea*[b]	*C. glutamicum*[c]			
	CjkS [*none*]	CjkR [*jk0412*]	K411 [*jk0412*]	CuR [*Cu1571*]	RES167 [*none*]	#3278 [*none*]	#3279 [*jk0412*]	#3322 [*Cu1571*]
Benzylpenicillin	1.5	>256	>256	>256	0.19	0.19	2(4+Sq[e])	2(4+Sq[e])
Benzylpenicillin/ clavulanate[d]	1.5	>256	>256	>256	<0.016	<0.016	2(4+Sq[e])	2(4+Sq[e])
Amoxicillin	1.5	>32	>32	>32	0.25	0.25	2(4+Sq[e])	2(4+Sq[e])
Imipenem	0.25	>32	>32	>32	0.094	0.094	0.5(2)	0.5(2)
Meropenem	0.25	1	>32	>32	0.03	0.03	0.5(2)	0.5(2)
Meropenem/ clavulanate[d]	0.25	>32	>32	>32	<0.002	<0.002	0.5(2)	0.5(2)
Ceftaroline	0.38	>256	>256	ND	ND	ND	ND	ND
Ceftobiprole	0.38	>32	>32	ND	ND	ND	ND	ND

The MICs were determined after 24 h of incubation. If an increase in the median (≥ 2-fold) of the MICs was detected after an additional incubation of 24 h, the median of the MICs observed at 48 h are indicated in brackets. ND: Not determined
[a]Values are the median of at least three determinations.
[b]*C. urea =C. urealyticum*
[c]*C. glutamicum #3278, #3279, and #3322* are derivatives of strain RES167 harboring plasmid pMM36, pMM36ΩP_{trc}-*jk0412* (pML2), and pMM36ΩP_{trc}-*cu1571*, respectively.
[d]Clavulanate 10 µg/mL
[e]Sq, presence of scatter colonies in the inhibition zone.

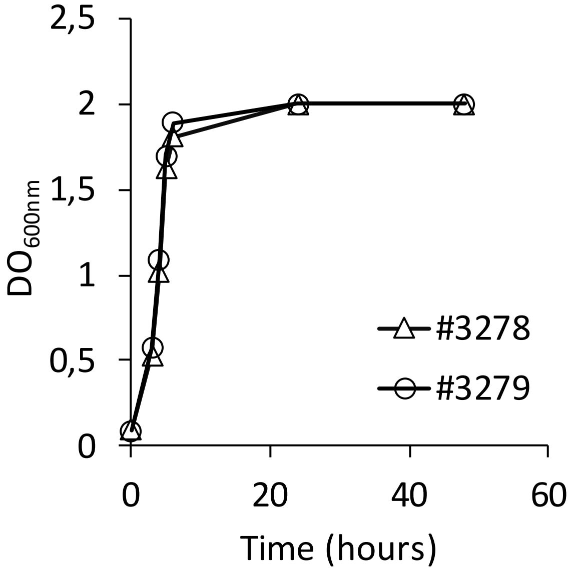

Figure S1. Impact of the Pbp2c protein on the growth of *C. glutamicum*. *C. glutamicum* strains. #3278 and #3279 are derivatives of *C. glutamicum* strain RES167 harboring plasmid pMM36 and pMM36ΩP_{trc}-*jk0412* (pML2), respectively. Strains were cultivated overnight without antibiotic, diluted to an OD_{600} of 0.1, and cultivated at 35 ± 2 °C for 48 hours without antibiotic.

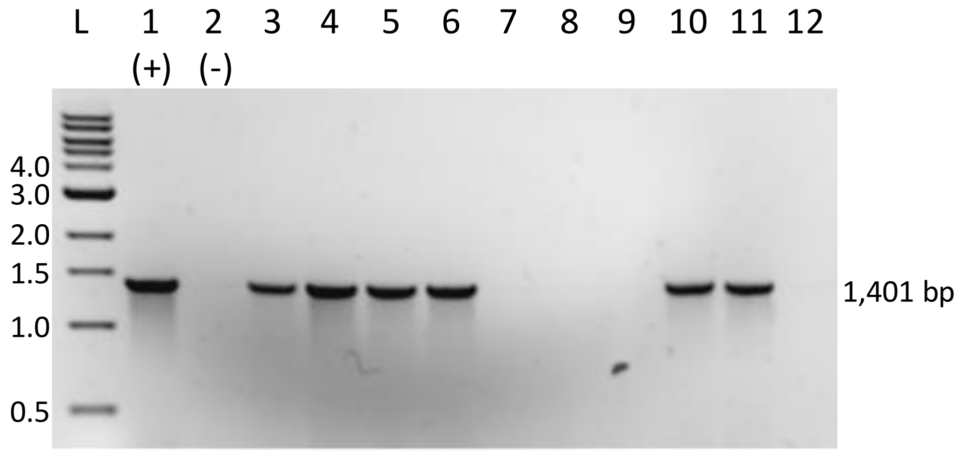

Figure S2. ***pbp2c* detection by PCR**. Five microliters of each 20 µL PCR reaction were loaded onto a 1% agarose gel supplemented with BET. Migration for 60 min at 90 Volts. L: 6 µL of 1kB ladder (NEB); Strains used in this experiment are described in Table 3: **1(+)**: #CjkR, **2(-)**: #Cjks, **3**: #CjkR-K411, **4**: #2023-ID6, **5**: #2023-1H6, **6**: #2023-2A3, **7**: #2023-1F5, **8**: #2023-2H3, **9**: #2023-4F2, **10**: #2023-6C6, **11**: #2023-6E7, **12**: #2017-1A2.

BlaC (*M. tuberculosis* β-lactamase, GenBank: ANZ82762.1)
MRNRGFGRRELLVAMAMLVSVTGCARHASGARPASTTLPAGADLADRFAELERRYDARLGVYVPATGTTAAIEYRADERFAFC**STFK**APLVAAVLHQNPLTHLDKLITYTSDDIRSISPVAQQHVQTGMTIG
QLCDAAIRYSDGTAANLLLADLGGPGGGTAAFTGYLRSLGDTVSRLDAEEPELNRDPPGDERDTTTPHAIALVLQQLVLGNALPPDKRALLTDWMARNTTGAKRIRAGFPADWKVID**KTG**TGDYGRANDIAV
VWSPTGVPYVVAVMSDRAGGGYDAEPREALLAEAATCVAGVLA

Translation product from *jk0411 (Bla$_{Cor}$)*
XXXXXXXXXXXXXXXXXXXXXXXXXXXXXXXXXXXXXXXMTARMEMALENVLTRSGCEGWWCASRLHDGVTIGRKQDEKITVA**SLYK**IRLLHLTLEAIQRGDLDPRLRVDVAPSKGTYEGYGFAAFDDAVNV
SLRDLMKQVASVSDNVAAHEILRLLPAWAREAFLKRFPPHYDLQDRNSLVTAEKQLRQYVQSQGGRSTDIAFSAVSSLAEITNDLEQVWKDDSAAVRRSMCELLGLQVWRHRVPSGFPPSGVDIYG**KTG**TVG
LLHGEASIIQVEGEEPIVVSIMLKQVVPEQAMSTHDGAIGEVARLLVDALR

Translation product from *jk0658*
MSSEVEDVLAELQDWPVDNVAAAALWGGETATFGDTSAQFPLASVSKLITAYATLLAVEEGAFELDQQVPAELLPEFDDLPTVRELLAHTSGISFRDRTPEKPRGTRRIYSSAGYEVLADFIAAEADMPFAD
YVAEGVCAPLGIEVKVEGSAGHGFSASVDALAVLCSEFLTPTVIAPSTLNDALEPQWPELSGVVPGYGMQKPCPWGLGFELHGEKSPHWLGEGMPESVAGHFGQSGTFLWIDRSGGNKKAAVVLTDENFGDW
AKQRWDGFNQRLWEALG

Translation product from *jk0584*
MTFNFSQYTYRPRHAAKDSRSAAKGIATAALTASIGVAGFAAPANAAPAGPPAGSQLPPLPQVQLPPAPQLPPLPPVPQAVEDALNQLGMSANQIPGSSAPQRPAPHRPAPNNGYKVRTIHNVPARTSMAVV
TNNGIAKTPNADEARPGLSIVKLYMADYVLRYGDHSNSDRYLTERMIRFSDDGAASKINRKYPRAISTIAREYGLRNTRAAAHWGNSYTSATDTALFLHNLRIRHPRSLVLHWMRTASPVAADGTRQDWGTV
HLPGVNGTKWGWSDYGRQTLASASFGNGYTVASFSWGGRGVQNADTRAAAGYLR

Translation product from *jk1553*
MPLFTSPRPGSVQRGSVRRGLVAAVVCAASVSVAGCTIGEVERDDANAPNPSTSAQPDGKPKSRGPQPTTGASTPEKPQEEQHGELGYNGATAIAVAGMDGAGETTVAPWSTAKVPVAIAALRKDPSVHGAM
TQAIQNSDNAAAETLWSSLGTPEQAAEATSAVIREGGSSARVESQEVRPGFSSFGQSDWSLSAQADFASHLRCIEGAEPVVSAMSNVGNGGGYGLGTIPGAIFKGGWGPDNAGAYGLRQFGLIPRSDGAGSG
YVAVAIAVESPDGSYESGQAELTAAAEFLRSKVDSLPKAAC

Translation product from *jk2026*
MASNERPEGASRRSQGWRGMYTGEPESQAGAQQGVTAGAQAADEQWGFASEPELQAGAQQGTAAMQADGSLAAEPDEPEGKPRNGARATRGARGGLVAGLAVGAAVLAITVGLVMGLSGGEGGVTKKGEESA
PAANTAAVPYTDGNDGANATPPTDEEQQQKLDKLAADIAEKNNVQVGVAIIGENGPLHAGKLNEEGAWSTIKVPIAGAVEEKLRHAEAHGQPAPRAAMEADMDAAIHYSDNDAAFRLWMYVGDGSDRTAAYK
VRDYMHRVGDPTNAAKQFEDGVYVGFGAIKWKLTDQVKFMNGFRCMNGSEKVLERMGHIIPEHKYGLAKINGAQFKGGWGPEPDGRFIYRQLGLVPGPDGEMTPVAIMAIPNDGLEPTAWDGVDALAKKLPD
VLEGAPPAKTKDDC

Figure S3. Translation products of the five *C. jeikeium* genes identified with similarity to β-lactamases and PBPs. The three conserved motifs SxxK, SDx and KTG are highlighted in green, red and yellow, respectively. The sequence of BlaC, the constitutive β-lactamase of *M. tuberculosis*, was incorporated for sequence comparisons with Bla$_{Cor}$. The first 39 amino acids of the BlaC protein are colored gray for comparison with the translation product of *jk0411* (Bla$_{Cor}$), the only protein found to have all three conserved β-lactamases motifs.

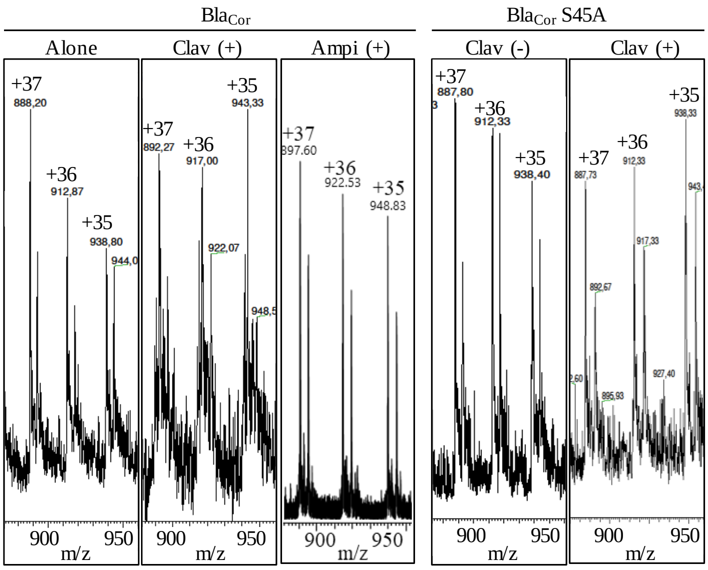

	Average mass	
	Calculated	Observed
Bla_{Cor}	32,825	32,825
Bla_{Cor} + Clav	32,980	32,982
Bla_{Cor} S45A	32,809	32,809
Bla_{Cor} S45A + Clav	32,964	32,807
Bla_{Cor} + Ampi	33,175	33,174

Figure S4. Formation of adducts between Bla_{Cor} with clavulanic acid and ampicillin and Bla_{Cor} S45A with clavulanic acid. Bla_{Cor} and Bla_{Cor} S45A were incubated without or with antibiotics. Peaks at m/z 888.20, 912.87, and 938.80 correspond to the $[M+37H]^{37+}$,$[M+36H]^{376+}$, and $[M+35H]^{35+}$, respectively. The additional peaks correspond to spontaneous α-*N*-6-phosphogluconoylation of the poly histidine tag (7). Mass spectrometry experiments were performed on an LCQ-Deca XP-Max instrument in positive mode. Proteins (5 µM) were incubated in presence or absence of antibiotics (50 µM) for 1 hour at room temperature. Prior to injection, proteins were desalted on a small Sephadex G-25 column equilibrated in ammonium acetate 5mM pH 6.4. Desalted proteins were quantified spectrophotometrically at 280 nm and injected into the apparatus at a flow rate of 10 µL/min.

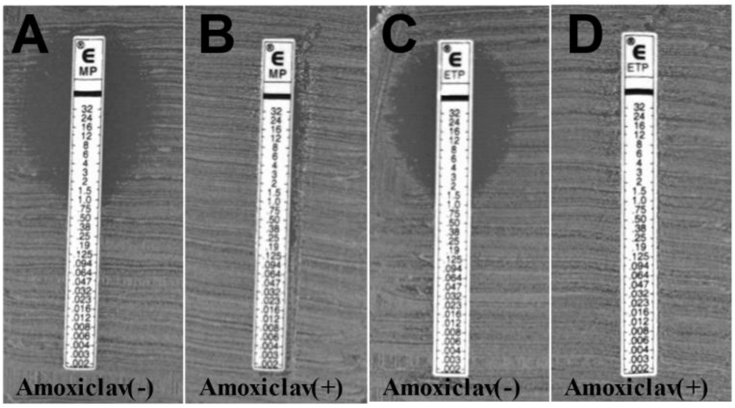

Figure S5. E-Test strip superimposition method for the detection of inducible β-lactam-resistant corynebacteria strains in the medical laboratory environment. Resistant *C. jeikeium* CIP103337 (CjkR) strain cultivated for 48 h on MHF medium. Meropenem strip without (**A**) or with (**B**) a 10 min preincubation with a strip containing amoxicillin-clavulanate (amoxiclav). Ertapenem strip without (**C**) or with (**D**) a 10 min preincubation with a strip containing amoxiclav.

REFERENCES

1. Tauch A, Kaiser O, Hain T, Goesmann A, Weisshaar B, Albersmeier A, et al. Complete Genome Sequence and Analysis of the Multiresistant Nosocomial Pathogen *Corynebacterium jeikeium* K411, a Lipid-Requiring Bacterium of the Human Skin Flora. J Bacteriol. 2005;187(13):4671-82.

2. Tauch A, Trost E, Tilker A, Ludewig U, Schneiker S, Goesmann A, et al. The lifestyle of *Corynebacterium urealyticum* derived from its complete genome sequence established by pyrosequencing. J Biotechnol. 2008;136(1-2):11-21.

3. Dusch N, Pühler A, Kalinowski J. Expression of the *Corynebacterium glutamicum* panD Gene Encoding L-Aspartate-α-Decarboxylase Leads to Pantothenate Overproduction in *Escherichia coli*. Appl Environ Microbiol. 1999;65(4):1530-9.

4. Brosius J. Expression Vectors Employing Lambda-, trp-, lac-, and lpp-Derived Promoters. In: Vectors. Elsevier; 1988. p. 205-25.

5. Houben K, Marion D, Tarbouriech N, Ruigrok RWH, Blanchard L. Interaction of the C-Terminal Domains of Sendai Virus N and P Proteins: Comparison of Polymerase-Nucleocapsid Interactions within the Paramyxovirus Family. J Virol. 2007;81(13):6807-16.

6. Merkamm M, Chassagnole C, Lindley ND, Guyonvarch A. Ketopantoate reductase activity is only encoded by ilvC in *Corynebacterium glutamicum*. J Biotechnol. 2003;104:253-60.

7. Geoghegan KF, Dixon HBF, Rosner PJ, Hoth LR, Lanzetti AJ, Borzilleri KA, et al. Spontaneous α-*N*-6-Phosphogluconoylation of a “His Tag” in *Escherichia coli*: The Cause of Extra Mass of 258 or 178 Da in Fusion Proteins. Anal Biochem. 1999;267:169-84.
